# Therapeutic Potential of Ginger against Renal Injury Induced by Carbon Tetrachloride in Rats

**DOI:** 10.1100/2012/840421

**Published:** 2012-04-01

**Authors:** Manal A. Hamed, Sanaa A. Ali, Nagy Saba El-Rigal

**Affiliations:** Therapeutic Chemistry Department, National Research Center, Dokki 12311, Cairo, Egypt

## Abstract

The objective of this study was to evaluate the potential of successive ginger extracts (petroleum ether, chloroform, and ethanol) against nephrotoxicity induced by CCl_4_ in rats. The evaluation was done through measuring kidney antioxidant parameters: glutathione (GSH), lipid peroxides (LPO), and superoxide dismutase (SOD). Renal function test: urea, creatinine and serum protein values, were also evaluated. The work was extended to examine tissue inflammatory mediators, prostaglandin-E_2_ (PGE_2_), collagen content and the kidney histopathology. Severe alterations in all biomarkers were observed after injury with CCl_4_. Treatment with ginger extracts resulted in markedly decreased levels of LPO, PGE_2_, collagen and kidney function tests, while increased levels of GSH, SOD and serum protein were observed. In conclusion, extracts of ginger, particularly the ethanol, resulted in an attractive candidate for the treatment of nephropathy induced by CCl_4_ through scavenging free radicals, improved kidney functions, inhibition of inflammatory mediators, and normalizing the kidney histopathological architecture. Further studies are required in order to identify the molecules responsible of the pharmacological activity.

## 1. Introduction

CCl_4_ is a potent, lipid-soluble hepatotoxin that, when bound to lipid and protein, enhances the peroxidative process [1]. Recent studies have demonstrated that CCl_4_ can cause generation of reactive oxygen species (ROS) in many tissues other than the liver including the kidney, heart, lung, testis, brain, and blood [2]. Free radicals that induce lipid peroxidation cause cell membrane damage leading to a number of pathological changes in acute and chronic renal injuries [3, 4]. The key enzyme involving CCl_4_-induced nephrotoxicity is cytochrome P450, which is localized in the cortical tubule cells, and the increased lipid peroxidation is evident in the renal brush border [5]. CCl_4_ also affects renal mitochondrial function including calcium flux across mitochondrial membranes [5].

Renal fibrosis is the principal process involved in the progression of chronic kidney disease [6], ureteral obstruction, malignant hypertension, severe diabetic condition, or chronic exposure to heavy metals [7]. The development of renal fibrosis involves the progressive appearance of glomerulosclerosis, tubulointerstitial fibrosis, and changes in renal vasculature [6]. At a molecular level, fibrosis can be defined as an excessive accumulation of extracellular matrices such as collagen and fibronectins [6]. The presence of kidney fibrosis seems mostly to be viewed as an endpoint or marker of tissue or organ failure and loss of function [8].

Reports documented that several herbal extracts and plant-derived pure molecules could protect organs against CCl_4_ by enhancing the antioxidant activity [9]. Endogenous antioxidants in medicinal herbs may play an important role as a defense against oxidative damage and protecting the biological functions of cells [10].

*Zingiber officinale* Roscoe (*Zingiberaceae*) is the most widely used spice worldwide. It has been reported as an antioxidant and detoxifying agent against alcohol abuse [11] and bromobenzene intoxication [12]. Islam and Choi [13] and Matsuda et al. [14] evaluated its antidiabetic and antihyperlipidemic activity. In addition, Habib et al. [15] showed its anticancer effect in liver hepatoma. Despite the favorable ethnopharmacological properties of ginger, its protective effect against nephrotoxicity by CCl_4_ has not previously been explored and its role as diminished factor of fibrosis could be a marker of therapeutic benefit.

 In the present study, we evaluated the effect of successive extracts of *Zingiber officinale *rhizome on nephropathy induced by CCl_4_. The evaluation was carried out through measuring antioxidant parameters, kidney function values, inflammatory mediators, and the histopathological architecture of the kidney.

## 2. Material and Methods

### 2.1. Chemicals

All chemicals in the present study were of analytical grade, products of Sigma (US), Merck (Germany), and BDH (England).

### 2.2. Plant Collection

*Zingiber officinale* was purchased from a local market (Hyper One Market, 6th October City, Egypt). The plant material was identified by Dr. Manal Shabana, Phytochemistry and Plant Systematic Department, National Research Center, Cairo, Egypt, and voucher specimen (ZOR-2010) was deposited as a reference. Dried rhizomes were ground in a grinder with 2 mm diameter mesh. Five hundred g of dry powder was kept in tightly closed container until needed.

### 2.3. Plant Extraction

The dried powered rhizome was sequentially extracted in a Soxhlet (Toshiba, India) apparatus using solvent of increasing polarities: petroleum ether (40–60°C), chloroform, and 95% ethanol for 72 h of each solvent [16]. Solvent removal was carried out under vacuum for drying at 40°C, producing semisolid residues of 1.3, 0.80, and 2.35% w/w, respectively.

### 2.4. Phytochemical Screening

All extracts were tested for sterols, tannins and terpenes [17], flavonoids [18], carbohydrates, and alkaloids [19].

### 2.5. Animals

Male Wistar albino rats (100 to 120 g) were selected for this study. They were obtained from the Animal House, National Research Center, Egypt. All animals were kept in controlled environment of air and temperature with access of water and diet *ad libitum*.

### 2.6. Ethics

Anesthetic procedures and handling with animals complied with the ethical guidelines of Medical Ethical Committee of the National Research Centre in Egypt (approval number: 10031).

### 2.7. Doses of Administration

Administration regime was twice a week for six consecutive weeks. Five hundred microliters of CCl_4_ diluted 1 : 9 (v/v) in olive oil was injected intraperitoneally [20]. Ginger extracts (200 mg/kg body weight) were administered orally [12]. Silymarin, a reference herbal drug (100 mg/kg body weight), was administered orally [21].

### 2.8. Experimental Design

60 male rats were used in this study. Animals were divided into 10 groups (6 rats each). Group 1 served as normal healthy control rats. Groups 2–5 were normal healthy rats orally administrated different ginger extracts (petroleum ether, chloroform, and ethanol) or silymarin. Group 6 was intraperitoneally injected with CCl_4_. Groups 7–9 were forced in the same time and for the same duration with CCl_4_ and different plant extracts. Group 10 was forced with CCl_4_ and silymarin.

### 2.9. Sample Preparations

Serum sample: blood was collected from each animal by puncture of sublingual vein in clean and dry test tubes, left 10 minutes at room temperature to clot, and centrifuged at 3000 rpm for serum separation. The separated serum was stored at −80°C for further determinations of kidney function tests and total protein.

 Tissue sample: kidney tissue was homogenized in cold 0.9 N NaCl (1 : 9 w/v) solution, centrifuged at 3000 rpm for 10 minutes, separated from the supernatant and stored at −80°C for further antioxidant determinations and prostaglandin-E_2_ (PGE_2_). 

### 2.10. Biochemical Assays

Lipid peroxide (LPO) was determined as malondialdehyde. Its concentration was calculated using the extinction coefficient value 1.56 < 10^5^ M^−1 ^cm^−1^ and read at 535 nm by the method of Buege and Aust [22].

 Glutathione (GSH) was assayed using dithiobis-2-nitrobenzoic acid (DTNB) in PBS according to Moron et al. [23]. The color developing reaction was read at 412 nm.

 Superoxide dismutase (SOD) was carried out by the method of Nishikimi et al. [24], where the oxidation of NADH was mediated by superoxide radical and the following increase in absorbance, measured at 560 nm using the molar extinction coefficient of NADH (6.22 × 10^3^ M^−1 ^cm^−1^).

 Urea was determined by the method of Tabacco et al. [25], where the conversion of urea in the sample by urease enzyme provided a colored complex that can be measured by spectrophotometry (LKB, Sweden) at 600 nm.

 Creatinine was measured by the method of Bartels and Böhmer [26]. Creatinine in the sample reacts with picrates in alkaline medium forming a colored complex at 500 nm.

 Serum total protein was assayed according to Bradford [27]. Coomassie Brilliant Blue dye reacts with Bradford reagent to give a blue complex, which is measured colorimetrically at 595 nm.

 PGE_2_ assay is based on the competition between PGE_2_ and PGE_2_-acetylcholinesterase conjugate for a limited amount of PGE_2_ monoclonal antibody. This antibody binds to goat polyclonal anti-mouse IgG attached previously to the well. After acetylcholinesterase substrate was added, a yellow color complex was formed and intensity of absorbance was read at 412 nm [28].

### 2.11. Histopathological Study

Kidney tissues were excised from sacrificed animals, individually weighed, and, from them, 5 *μ*m thickness slices were cut, fixed in 10% paraformaldehyde, and embedded in paraffin wax blocks. Tissue sections of 5 *μ*m thick were stained with haematoxylin and eosin (H&E) and Masson's trichrome and then examined under light microscope for determination of pathological changes. A minimum of 10 fields for each slide were examined and scored semiquantitatively for severity of changes and collagen deposition. The scoring was done as none (−), mild (+), moderate (++), and severe (+++) changes. Collagen deposition (blue spots) was expressed as normal (±10%), mild (10–25%), moderate (26–50%), and marked (>56%) [29].

### 2.12. Statistical Analysis

All data were expressed as mean ± SD of six rats in each group. Statistical analysis was carried out by one-way analysis of variance (ANOVA), Costat Software Computer Program:

(1) $$\begin{array}{cc} \text{\%}\;\text{change} & =\frac{\text{control}\;\text{mean}-\text{treated}\;\text{mean}}{\text{control}\;\text{mean}}\times 100,\\ \text{\%}\;\text{improvement} & =\frac{\text{treated}\;\text{mean}-\text{intoxicated}\;\text{mean}}{\text{control}\;\text{mean}}\times 100. \end{array}$$

## 3. Results and Discussion

### 3.1. Phytochemical Screening

Our previous work by [30] revealed the presence of lipid contents in petroleum ether extract. Chloroform extract contained moderate concentrations of sterols and terpenes. Phytochemical screening of the ethanol extract revealed abundant presence of flavonoids and tannins. High concentration of carbohydrates and moderate concentration of alkaloids were also recorded. These data were in accordance with Anosike et al. [31] who found the same major constituents in ginger ethanol extract. It was clear that the concentrations of the most active compounds were present in ethanol extract.

### 3.2. Potency of Ginger as Free Radicals Scavenger

**Figure 1:**
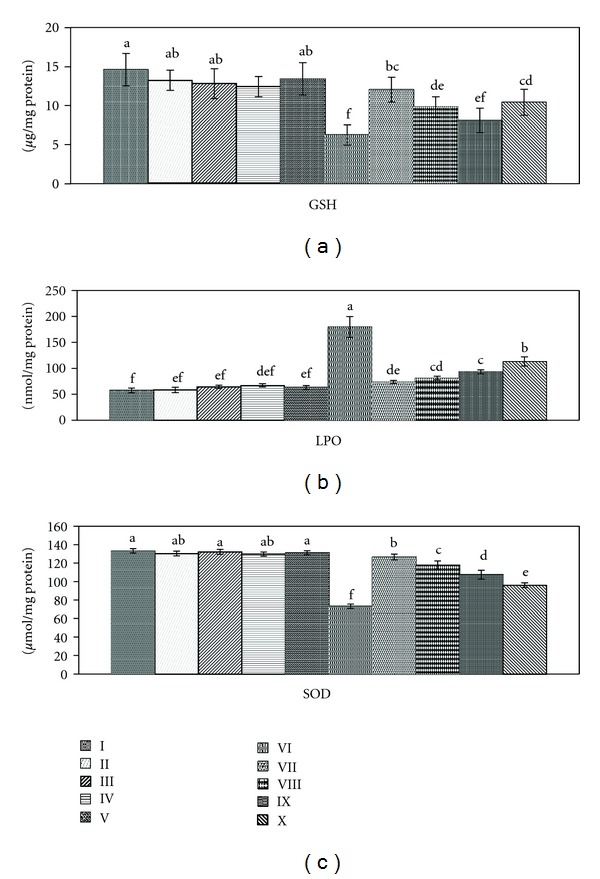
Effect of *Zingiber officinale* on antioxidant levels: glutathione (GSH), lipid peroxides (LPO) and superoxide dismutase (SOD). Groups I: control, II: control treated with ethanol extract, III: control treated with chloroform extract, IV: control treated with petroleum ether extract, V: control treated with silymarin, V1: intoxicated with CCl_4_, VII: intoxicated with CCl_4_ and treated with ethanol extract, VIII: intoxicated with CCl_4_ and treated with chloroform extract, IX: intoxicated with CCl_4_, treated with petroleum ether extract, and X: intoxicated with CCl_4_ and treated with silymarin. Data are mean ± SD of six rats in each group. Statistical analysis is carried out by one-way analysis of variance (ANOVA); Costat Computer Program. Unshared letters are significance values between groups at *P* < 0.05.

Some chemicals cause damage to renal tissue by ROS production. CCl_4_ is known to induce ROS, deplete antioxidant defenses, and lead to oxidative stress in different tissues. With regard to the enzymatic and nonenzymatic antioxidant levels, the present study revealed significant reduction in glutathione (57.53%) and superoxide dismutase (54.04%) in CCl_4_-treated rats, while lipid peroxides recorded significant increase (212.26%, Figure 1). This observation was in accordance with Khan et al. [4] who reported the same disturbance in the antioxidant levels after CCl_4_ induction of damage. The reduction of glutathione level concerning its role as antioxidant gives an additional support of the elevation of free radicals involved in renal toxicity by CCl_4_. In addition, reductive dehalogenation of CCl_4_ by the P450 enzyme system to the highly reactive trichloromethyl radical initiates the process of lipid peroxidation, which is considered to be the most important mechanism in the pathogenesis of renal damage [3]. Moreover, these metabolites can react with sulfhydryl groups of glutathione and protein thiols to alter the redox status of cells [32]. Therefore, GSH is considered an important defense against lipid oxidative damage in the kidneys eliminating hydrogen peroxide, peroxyl and hydroxyl radicals formed during this process. As SOD is a glutathione-level-dependent enzyme, its activity was decreased by the depletion of glutathione level [33]. Hence, it was identified as a potential urinary marker of CCl_4_-induced hepatorenal toxicity [34].

 Treatment with ethanol extract of ginger recorded improvement of the GSH, LPO, and SOD by 39.72, 184.01, and 40.09%, respectively. Chloroform extract enhanced the antioxidant parameters by 24.65, 171.15, and 33.48%, while petroleum ether showed amelioration with 12.73, 150.29 and 25.67%. It was clear that ethanol extract recorded the highest improvement levels due to its higher concentration of flavonoids, tannins, and alkaloids, the naturally occurring antioxidants. Silymarin as hepatic supportive drug may also play a role in attenuation of free radicals deleterious action on the kidney through stimulation of the antioxidant efficiency by 28.76, 115.53, and 17.19%, respectively. Preventive effects of ginger against CCl_4_-induced oxidative stress could be attributed to its high level of polyphenol compounds (6-gingerol and its derivatives), which have a high antioxidant activity [35]. These compounds could scavenge the free radicals of CCl_4_ generated through P450 enzyme system and thereby diminish the oxidative injuries. Ginger may also impair CCl_4_-mediated lipid peroxidation through decreased production of free radical derivatives. Administration of ginger extracts to Group 1 animals showed insignificant changes in all the antioxidant parameters confirming the potency of the ginger extracts as anti-free-radicals-producer.

### 3.3. Effect of Ginger on Renal Disorder Biomarkers

**Figure 2:**
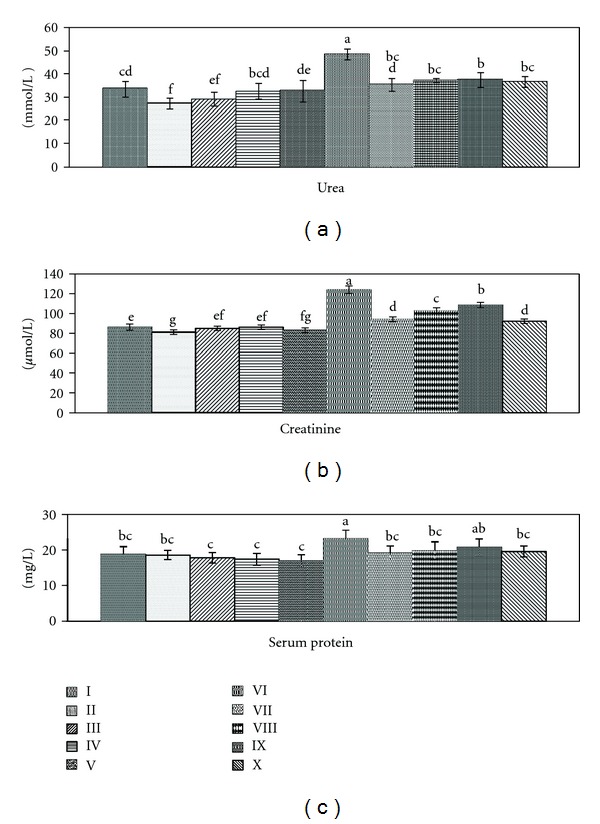
Effect of *Zingiber officinale* on kidney function parameters: urea, creatinine, and serum protein. Groups I: control, II: control treated with ethanol extract, III: control treated with chloroform extract, IV: control treated with petroleum ether extract, V: control treated with silymarin, V1: intoxicated with CCl_4_, VII: intoxicated with CCl_4_ and treated with ethanol extract, VIII: intoxicated with CCl_4_ and treated with chloroform extract, IX: intoxicated with CCl_4_ and treated with petroleum ether extract, and X: intoxicated with CCl_4_ and treated with silymarin. Data are mean ± SD of six rats in each group. Statistical analysis was carried out by one-way analysis of variance (ANOVA); Costat Computer Program. Unshared letters are significance values between groups at *P* < 0.05.

High levels of urea (44.60%), creatinine (43.70%), and lower level of serum protein (23.40%) were recorded in CCl_4_ group (Figure 2). This was in agreement with Khan et al. [3] who reported that chronic renal injuries by CCl_4_ intoxication were associated with urea and creatinine elevation and considered as indicators of kidney injury, where the serum creatinine level does not rise until at least half of the kidney nephrons are destroyed. Renal injuries may contribute to low level of serum protein that might have resulted from remarkable leakage into urine due to injuries in glomeruli and tubules [4].

 Treatment with different extracts of ginger ameliorated kidney function parameters by 39.52, 34.55, and 21.27% for ethanol extract and 33.49, 24.35, and 18.08% for chloroform extract, while petroleum ether showed improvement by 32.86, 17.86, and 12.76% for urea, creatinine, and serum protein, respectively. Silymarin ameliorated the kidney function markers by 35.24, 36.87, and 19.14%. Some investigators documented that different plant extracts significantly improve renal injuries induced by CCl_4_ intoxication [3, 36]. Normal rats treated with ginger extracts recorded significant decrease in urea and creatinine levels as compared to control group (Group 1), which give an additional support to the role of the extracts in eliminating waste products and as antioxidant. This findings were in agreement with Mehrdad et al. [37] who stated that ginger has a beneficial effect for removal of urea and creatinine from plasma of normal mice treated with its alcoholic extract and considered as a therapeutic herb to manage renal function.

### 3.4. Role of Ginger against Inflammation

**Figure 3:**
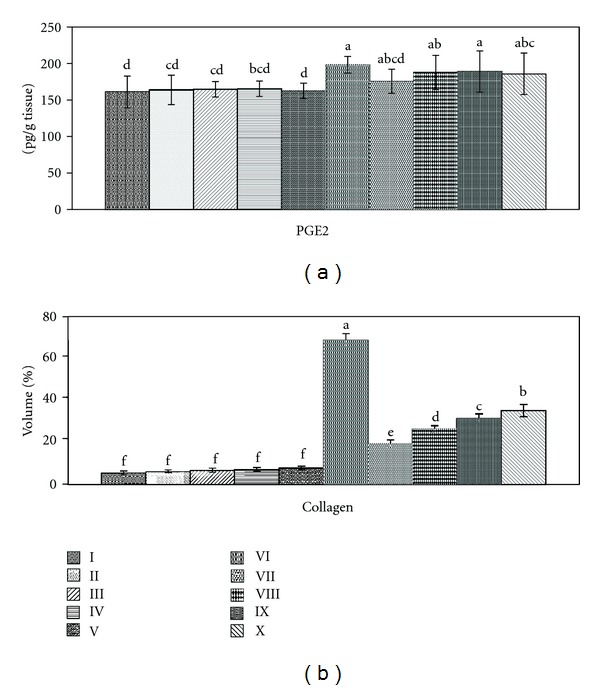
Effect of *Zingiber officinale* on prostaglandin E_2_ (PGE_2_) and collagen percentage. Groups I: control, II: control treated with ethanol extract, III: control treated with chloroform extract, IV: control treated with petroleum ether extract, V: control treated with silymarin, V1: intoxicated with CCl_4_, VII: intoxicated with CCl_4_ and treated with ethanol extract, VIII: intoxicated with CCl_4_ and treated with chloroform extract, IX: intoxicated with CCl_4_ and treated with petroleum ether extract, and X: intoxicated with CCl_4_, treated with silymarin. Data of PGE_2_ are mean ± SD of six rats in each group. Collagen percentages are mean of ten fields of collagen deposition under light microscope (100x). Statistical analysis is carried out by one-way analysis of variance (ANOVA); Costat Computer Program. Unshared letters are significance values between groups at *P* < 0.05.

In kidney, PGE_2_ is the main prostaglandin, playing important roles in vasoconstriction, maintaining homeostasis, development of pathological settings, and regulation of salt and water reabsorption. PGE_2_, as an inflammatory mediator, recorded significant increase (23.11%) in kidney tissue of CCl_4_-treated group (Figure 3). This was in accordance with Choi et al. [38] who recorded PGE_2_ enhancement after acute nephrotoxicity of melamine, cyanuric acid, and a mixture of them. Carlsen et al. [39] observed severe production of PGE_2_ in renal medullary interstitial cells when subjected to osmotic, inflammatory, and mechanical stress. Oxidative stress induced by CCl_4_ can promote renal vasoconstriction [4], which enhanced PGE_2_ production. Nørregaard et al. [40] added that bilateral ureteral obstruction promoted accumulation of prostaglandins in cortex and inner medullar tissue of kidney in rats.

Treatment with ginger extracts (ethanol, chloroform, and petroleum ether) improved PGE_2_ levels by 14.05, 6.52, and 5.75% respectively, while silymarin recorded 7.70%. This was in accordance with Hsu et al. [41] who found that* Orthosiphon aristatus*, used for the treatment of renal inflammation, kidney stones, and dysuria, would act by suppression of the production of lipopolysaccharide-induced nitric oxide and PGE_2_ by inhibiting ROS generation and reducing the expression of inducible nitric oxide synthase and cyclooxygenase-2.

### 3.5. Efficacy of Ginger against Glomerular Sclerosis

The normal glomerular basement membrane, composed of type IV collagen, has an important function in the process of filtration [42]. Therefore, increased collagen production by mesangial cells plays a key role in the development and progression of glomerular sclerosis [43]. These data were in parallel with our results by the observed glycogen deposition (60.2%, Figure 3) in kidney after CCl_4_ induction. Treatment with different ginger extracts and silymarin recorded collagen deposition by 17, 23.2, 27.6, and 30.8% respectively. Normal rats treated with plant extracts and silymarin showed normal levels of collagen (5.4, 5.8, 6.2, and 6.8%, resp.), revealing a role of the extract as a non inducer of collagen deposition or fibrosis.

### 3.6. Ginger against Nephropathy Induced by CCl_4_

**Figure 4:**
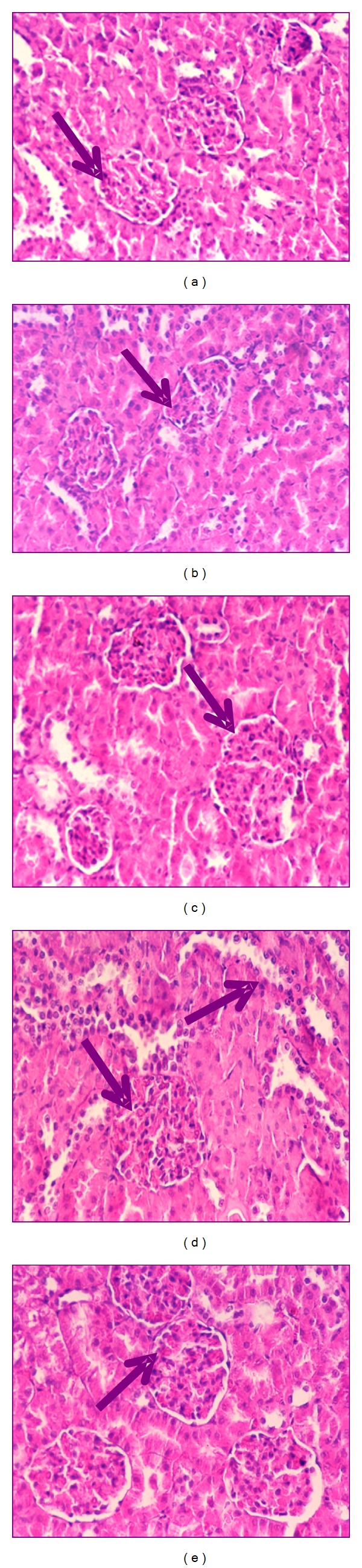
Photomicrography of kidney sections (200x) of control (a), control treated with ethanol extract (b), control treated with chloroform extract (c), control treated with petroleum ether extract (d), and control treated with silymarin (e) stained with haematoxylin and eosin.

**Figure 5:**
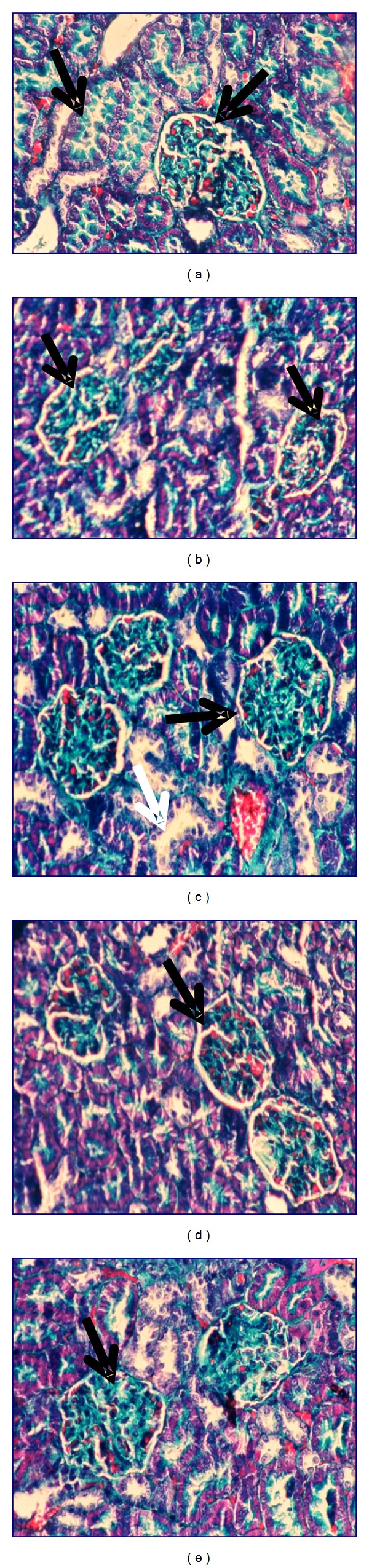
Photomicrography of kidney sections (200x) of control (a), control treated with ethanol extract (b), control treated with chloroform extract (c), control treated with petroleum ether extract (d), and control treated with silymarin (e) stained with Masson's trichrome.

 Kidney histopathological features of control and control treated rats with ethanol, chloroform, petroleum-ether extracts, and silymarin showed normal appearance of tubules, glomeruli, and tubulointerstitial cells (Figures 4(a), 4(b), 4(c), 4(d), and 4(e), resp.). Collagen deposition was of normal range in all control groups (Figures 5(a), 5(b), 5(c), 5(d), and 5(e), resp.).

**Figure 6:**
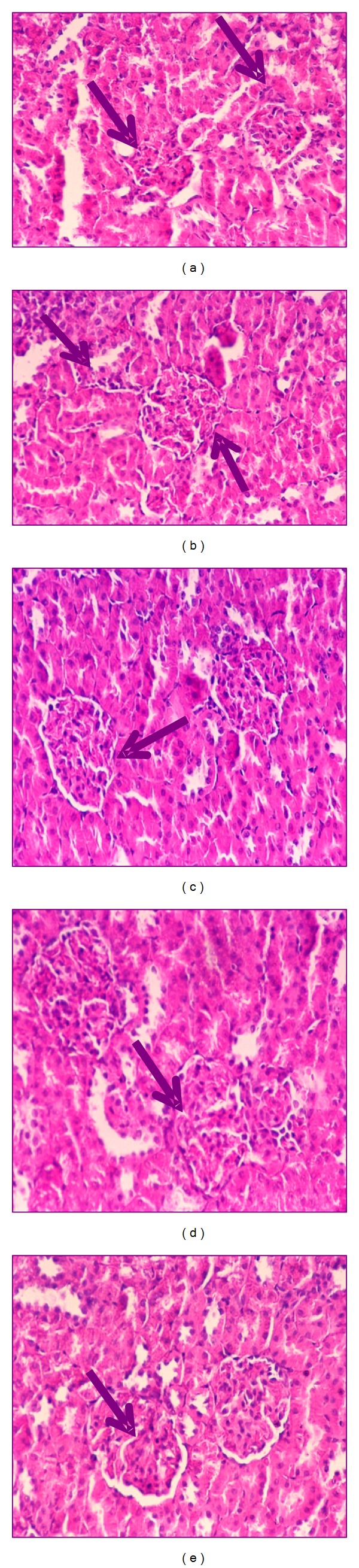
Photomicrography of kidney sections (200x) of CCl_4_ (a), CCl_4_ treated with ethanol extract (b), CCl_4_ treated with chloroform extract (c), CCl_4_ treated with petroleum ether extract (d), and CCl_4_ treated with silymarin (e) stained with haematoxylin and eosin.

**Figure 7:**
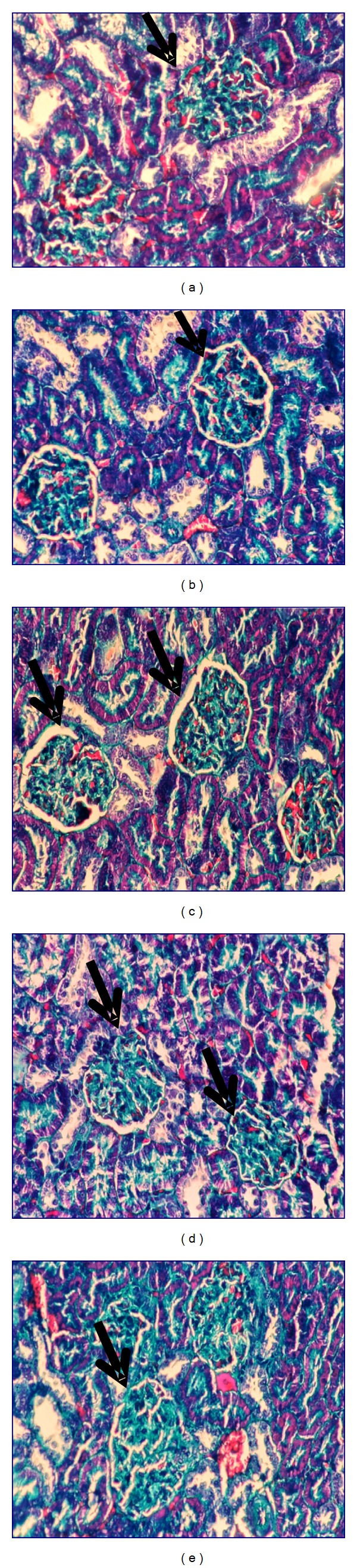
Photomicrography of kidney sections (200x) of CCl_4_ (a), CCl_4_ treated with ethanol extract (b), CCl_4_ treated with chloroform extract (c), CCl_4_ treated with petroleum ether extract (d), and CCl_4_ treated with silymarin (e) stained with Masson's trichrome.

 Kidney section of CCl_4_-treated rats showed glomerular and tubular degenerations varying from glomerular basement membrane thickening, mild dilatation or congestion of space of Bowman, interstitial inflammation, tubular cell swelling or congestion, tubular brush border loss, tubular dilatation, and necrosis of epithelium to interstitial oedema (Figure 6(a)). Marked collagen deposition was recorded (60.2%, Figure 7(a)).

 Ozturk et al. [44] recorded similar histopathological alterations in rats kidney treated with CCl_4_ characterized by tubular epithelial cells alterations including vacuolization, atrophy, detachment of epithelial cells, and tubular necrosis. With these histopathological changes, the capacity of tubular absorption may have been altered and functional overloading of nephrons with subsequent renal dysfunction was observed [3].

 Carbon tetrachloride group treated with ethanol extract of ginger showed almost normal morphology and normal architecture of the kidney (Figure 6(b)). Glomeruli and tubules appeared to be regenerated following chloroform extract (Figure 6(c)). Group of CCl_4_ treated with petroleum ether extract showed normal morphology with the exception of only few swollen glomeruli and rare vascular congestions that were present in both cortical and corticomedullar regions (Figure 6(d)). Kidney section of CCl_4_ treated with silymarin revealed regeneration of renal cells and regeneration of tubules nearly as seen in control group (Figure 6(e)). In all treated groups, neither interstitial inflammatory cell infiltrations nor an increase in the connective tissue cells was observed. Mild collagen deposition in ethanol- and chloroform-treated groups was observed (17.00 and 23.20%, resp.; Figures 7(b) and 7(c)), while moderated deposition was recorded after treatment with petroleum ether extract and silymarin of 27.60 and 30.80%, respectively (Figures 7(d) and 7(e)). The histopathological changes were graded and summarized in Table 1.

 The corrective histopathological findings after treatment with ginger extracts give an additional support that ginger mops up free radicals generation by CCl_4_, reduces inflammation, improves kidney function, and induces healthy state of renal cells, suggesting its role as renal protective agent. This was attributed to their abundance of natural antioxidants: flavonoids, sterols, and alkaloids. Also, silymarin, as an antioxidant flavonoid complex derived from the herb milk thistle (*Silybum marianum*), has the ability to scavenge free radicals, chelate metal ions, and inhibit lipid peroxidation [44]. In parallel with our results, ginger recorded a promise role against renal ischemia [45], renal damage induced by alcohol intoxication [46], and renal toxicity induced by doxorubicin, the commonly used anticancer drug [47].

## 4. Conclusions

CCl_4_-induced nephrotoxicity by free radicals involvement impaired renal functions, altered antioxidant levels, enhanced inflammation and fibrosis, affecting harmfully the kidney functionality. Ginger provides evidence for kidney protection and reduces severity of damage induced by CCl_4_ intoxication. Ethanol extract recorded the most potent effect due to its content of flavonoids, sterols, triterpenes, carbohydrate, and alkaloids. However, further detailed studies are required to establish its clinical application and to identify the molecules responsible of the pharmacological activity.

## Figures and Tables

**Table 1:** Effect of petroleum ether, chloroform, ethanol extracts, and silymarin drug treatment on histopathological examination of rats kidney exposed to CCl_4_.

Groups	Tubular cell swelling	Interstitial inflammation	Tubular dilatation	Necrosis of epithelium	Glomerular Hypercellularity
Control	—	—	—	—	—
CCL_4_	+++	+++	+++	+++	+++
CCl_4_ + ethanol extract	—	—	—	—	—
CCl_4_ + chloroform extract	+/–	+/–	—	—	+/–
CCl_4_ + petroleum-ether extract	+/–	++	+/–	+/–	+
CCl_4_ + sylimarin drug	—	—	—	—	—

Severity of renal histological changes using scores on a scale of none (–), mild (+), moderate (++), and severe (+++) damage.
